# Differential diagnosis between hepatic alveolar echinococcosis and intrahepatic cholangiocarcinoma with conventional ultrasound and contrast-enhanced ultrasound

**DOI:** 10.1186/s12880-020-00499-8

**Published:** 2020-08-27

**Authors:** Zeng-Cheng Wa, Ting Du, Xian-Feng Li, Hui-Qing Xu, Qiu-Cuo Suo-Ang, Li-Da Chen, Hang-Tong Hu, Wei Wang, Ming-De Lu

**Affiliations:** 1 Department of Medical Ultrasonics, Qinghai Red Cross Hospital, Xining, China; 2 Department of Medical Ultrasonics, People’s Hospital of Chengduo County, Yushu Prefecture, China; 3 Department of Medical Ultrasonics, Ultrasomics Artificial Intelligence X-Lab, Institute of Diagnostic and Interventional Ultrasound, The First Affiliated Hospital of Sun Yat-Sen University, 58 Zhongshan Road 2, Guangzhou, 510080 People’s Republic of China; 4 Department of Hepatobiliary Surgery, The First Affiliated Hospital of Sun Yat-Sen University, Guangzhou, China

**Keywords:** Ultrasonography, Contrast-enhanced ultrasound, Echinococcosis, Cholangiocarcinoma, Diagnosis

## Abstract

**Background:**

Misclassifications of hepatic alveolar echinococcosis (HAE) as intrahepatic cholangiocarcinoma (ICC) may lead to inappropriate treatment strategies. The aim of this study was to explore the differential diagnosis with conventional ultrasound and contrast-enhanced ultrasound (CEUS).

**Methods:**

Sixty HAE lesions with 60 propensity score-matched ICC lesions were retrospectively collected. The 120 lesions were randomly divided into a training set (*n* = 80) and a testing set (*n* = 40). In the training set, the most useful independent conventional ultrasound and CEUS features was selected for differentiating between HAE and ICC. Then, a simplified US scoring system for diagnosing HAE was constructed based on selected features with weighted coefficients. The constructed US score for HAE was validated in both the training set and the testing set, and diagnostic performance was evaluated.

**Results:**

Compared with ICC lesions, HAE lesions were mostly located in the right lobe and had mixed echogenicity, a pseudocystic appearance and foci calcifications on conventional ultrasound. On CEUS, HAE lesions showed more regular rim-like enhancement than ICC lesions and had late washout with a long enhancement duration. The simplified US score consisted of echogenicity, pseudocystic/calcification, bile duct dilatation, enhancement pattern, enhancement duration, and marked washout. In the testing set, the sensitivity, specificity, LR+, LR- and the area under the ROC curve for the score to differentiate HAE from ICC were 80.0, 81.3%, 4.27, 0.25 and 0.905, respectively.

**Conclusions:**

The US score based on typical features from both conventional ultrasound and CEUS could accurately differentiate HAE from ICC.

## Background

Alveolar echinococcosis (AE) is a globally distributed parasitic disease caused by an infection of Echinococcus multilocularis [1, 2]. The invasive growth pattern of AE resembles that of malignancy, and AE is acknowledged as one of the world’s most lethal chronic parasitic conditions [3, 4]. Hepatic alveolar echinococcosis (HAE), AE at its most frequently involved site, constantly invades intrahepatic vessels, bile ducts and hilum with no clear histological margin between the parasitic tissue and the adjacent normal liver parenchyma [5]. HAE should be differentiated from other benign or malignant focal liver lesions, such as hemangioma, hepatapostema, and especially intrahepatic cholangiocarcinoma (ICC) [6]. As indicated by Stojkovic et al., the treatment decisions of 26/80 patients with HAE were based on an incorrect diagnosis [6]. Infiltrative HAE was commonly confused with ICC [6, 7]. These misclassifications of HAE as ICC may lead to the determination of inappropriate treatment strategies that are potentially harmful for patients.

Although the clinical significance of misclassifying HAE as ICC has been raised recently, the differential diagnosis has not been extensively studied. A study by Mueller et al. found that no or septal enhancement and matrix calcifications on Computed Tomography (CT) and Magnetic Resonance Imaging (MRI) offered the strongest discriminating potential between HAE and ICC with a high sensitivity and specificity [7]. Ultrasound (US) is accepted as the first-choice imaging modality in the diagnosis and follow-up for patients suspected to have HAE. Conventional ultrasound is extensively used, but contrast enhanced ultrasound (CEUS) has been implemented in clinical practice for the diagnosis of HAE [8–10] and can visualize the parenchymal microvasculature to provide more information for differential diagnoses. Since our first study on ICC in 2008, we have reported specific features of ICC for differentiate it from other liver lesions [11–13]. The aim of this study was to compare the imaging features of HAE and ICC both on conventional ultrasound and CEUS and to establish a diagnostic US score for HAE. To the best of our knowledge, no study has been focused on this topic.

## Methods

### Patients

Between January 2017 and March 2018, 57 patients with HAE with 60 lesions were retrospectively collected at the ChengDuo County Hospital (*n* = 43) and QingHai Red Cross Hospital (*n* = 14), which are located in the major endemic region of Qinghai Province. The diagnosis for HAE was based on the criteria established by the World Health Organization Informal Working Group on Echinococcosis (WHO-IWGE) [3], and patients were included in this study if they had the following: 1) clinical and epidemiological history of living in pastoral areas with HAE, 2) conventional ultrasound and CEUS images, and 3) histologically or clinically proven HAE.

One hundred seventy patients with 170 ICC lesions were retrospectively collected at the First Affiliated Hospital of Sun Yat-sen University from January 2015 to March 2018. The inclusion criteria were patients with histologically proven ICC with integrated conventional ultrasound and CEUS images.

Propensity score matching was used to adjust for selection bias and to control for potential differences in the characteristics of patients. The variables for matching were sex and tumor size. HAE and ICC lesions were matched 1:1 using a three-digit matching algorithm with the nearest modality. Finally, 60 HAE lesions and 60 ICC lesions were analyzed in this study. There were 26 men and 34 women who were aged 38.9 ± 14.2 (mean ± S.D.) years (range, 12–77 years) for HAE and 26 men and 34 women who were aged 58.5 ± 9.6 (mean ± S.D.) years (range, 39–82 years) for ICC.

### Conventional ultrasound and contrast-enhanced ultrasound

Ultrasound examinations were performed using Logiq S7 (GE Healthcare, Little Chalfont, UK) or ProSound F37 (Hitachi Medical Systems, Tokyo, Japan) at ChengDuo County Hospital or QingHai Red Cross Hospital. In the First Affiliated Hospital of Sun Yat-sen University, the Aplio 500 scanner (Toshiba Medical Systems, Tokyo, Japan) equipped with a 375BT convex transducer (frequency, 3.5 MHz) was used. Before the CEUS, a baseline grayscale ultrasound was performed to scan the entire liver. The imaging settings of the ultrasound scanner were optimized to obtain the best depiction of the target lesion. The diagnostic information including the diameter, echogenicity, shape, and margin of each lesion, was recorded. The contrast-specific imaging modes used in the present study were under a mechanical index of 0.07–0.12. After activating the contrast-specific imaging mode, 2.4 ml of SonoVue (Bracco, Milan, Italy) was administered intravenously in a bolus fashion and flushed with 5 ml 0.9% saline solution. The target lesion was observed continuously for 4–6 min, and the entire arterial and portal phases and several repetitions of the late phase were stored on the hard disk. The arterial, portal and late phases were defined as 0–40 s, 41–120 s and 121–360 s after the injection, respectively. All US examinations were performed by two experienced radiologists (W.Z.C. and D.T., each with more than 4 years of experience in liver CEUS). The digital cine clips of the conventional ultrasound and the entire CEUS examination were stored on a hard disk incorporated in the scanner, and the image files were transferred to a removable disk for subsequent analysis. The data disk was sent to the ultrasound department of the First Affiliated Hospital of Sun Yat-Sen University for further data exploration.

### Image analysis

Two experienced radiologists (C.L.D. and W.W. with at least 8 years of experience in liver CEUS) randomly reviewed all the cine loops offline on screen in consensus. Both readers were not involved in the original examinations and were asked to document the following characteristic signs of lesion on conventional ultrasound: location, size, echogenicity, pseudocystic appearance, calcification of the lesion, and bile duct dilatation (diameter of intra-hepatic bile duct > 3 mm, or extra-hepatic bile duct > 8 mm).

The characteristic signs on CEUS were evaluated as follows [13–15]: a) enhancement level -- hyper-, iso- or hypoenhancing relative to the adjacent normal liver parenchyma; b) no enhancement -- no appearance of microbubble signals in the lesion; c) heterogeneous enhancement -- lesion enhancement with a different level of echogenicity; d) regular rim enhancement -- microbubble signals detected at a regular peripheral portion of the lesion; e) irregular rim enhancement -- microbubble signals detected at an irregular peripheral portion of the lesion; f) enhancement start time, washout time, and enhancement duration; g) lesion shape and margin; and h) washout level -- mild, moderate, or marked washout relative to the adjacent liver parenchyma.

### Statistical analysis

Statistical analysis and the random sequence numbers were performed by using SPSS 16.0 software (SPSS Inc., Chicago, IL, USA) and R software (R Foundation for Statistical Computing, version 3.2.5, http://www.r-project.org/, Austria). Data are presented as the mean ± standard deviation (SD) and percentage (%). *P* < 0.05 was considered to indicate statistical significance. The association between lesion size and imaging features was assessed using χ^2^ or Fisher’s exact tests. Student’s t-test was used to compare lesion size within the same characteristic imaging findings.

The 120 lesions were randomly divided into a training set (*n* = 80) and a testing set (*n* = 40). In the training set, the method of least absolute shrinkage and selection operator (LASSO) regularized regression was used to select the most useful independent conventional ultrasound and CEUS features for differentiating between HAE and ICC, respectively. Then, a US scoring system for diagnosing HAE that including features from both conventional ultrasound and CEUS was constructed for each patient using a linear equation of the combined selected features that were weighted by their respective coefficients. The constructed US scoring system for HAE was validated in both the training set and the testing set. The receiver operating characteristic (ROC) curve for differentiating between HAE and ICC was analyzed by calculating the area under the curve (AUC). The ROC curve was plotted to demonstrate the diagnostic performance of CEUS in the testing set. Sensitivity, specificity, positive predictive value (PPV), and negative predictive value (NPV) were calculated with an optimal cutoff value that maximized the sum of sensitivity and specificity.

## Results

### Conventional ultrasound features for HAE and ICC

All HAE and ICC lesions were confirmed by pathologic evaluation after surgery. Ten (16.7%) of the 60 observed HAE lesions were located in the left lobes of the livers, and 50 (83.3%) were in the right lobes. There were 27 (27/60, 45.0%) and 33 (33/60, 55.0%) ICC lesions located in the left and right lobes, respectively (*P* = 0.002). Forty-three (71.7%) HAE lesions showed mixed echogenicity, and only 7 HAE lesions showed hypoechogenicity; 34 (56.7%) ICC lesions showed hypoechogenicity. Only 2 (3.3%) HAE lesions were accompanied by bile duct dilatation in the liver, but 21 (35.0%) ICC lesions were accompanied by bile duct dilatation (*P* = 0.000). A pseudocystic appearance was observed for 14 (23.3%) HAE lesions but only for 1 (1.7%) ICC lesion. Notably, foci calcifications were observed in 29 (48.3%) HAE lesions but not in any ICC lesions (*P* = 0.000). Details of the HAE lesions and ICC lesions in the control group are listed in Table 1. Statistical analyses demonstrated that there were no significant differences between the training set and testing set for all US features (Table 2).

**Table 1:** Characteristics on US between HAE and ICC

Imaging Features on US	Total(***n*** = 120)		P*
	HAE (*n* = 60)	ICC (*n* = 60)	
**Conventional US**			
***Location***			0.002
Right lobe	50 (83.3)	33 (55.0)	
Left lobe	10 (16.7)	27 (45.0)	
***Lesion size***			0.851
≤ 5.0 cm	22 (36.7)	24 (40.0)	
> 5.0 cm	38 (63.3)	36 (60.0)	
***Echogenicity***			0.000
Hyper or Iso	10 (16.7)	7 (11.6)	
Mixed	43 (71.7)	19 (31.7)	
Hypo	7 (11.6)	34 (56.7)	
***Margin***			1.000
Clear	28 (46.7)	28 (46.7)	
Unclear	32 (53.3)	32 (53.3)	
***Bile duct dilatation***	2 (3.3)	21 (35.0)	0.000
***Pseudocystic appearance***	14 (23.3)	1 (1.7)	0.000
***Calcification***	29 (48.3)	0 (0)	0.000
**CEUS**			
***Arterial phase***			0.008
Hyper-	39 (65.0)	50 (83.3)	
Iso-	10 (16.7)	9 (15.0)	
Hypo- or Non-	11 (18.3)	1 (1.7)	
***Enhancement pattern***			0.000
Regular rim	53 (88.3)	6 (10.0)	
Irregular rim	6 (10.0)	29 (48.4)	
Heterogeneous	1 (1.7)	14 (23.3)	
Homogeneous	0 (0)	11 (18.3)	
***Portal phase***			0.000
Hyper-	1 (1.7)	0 (0)	
Iso-	19 (31.6)	1 (1.7)	
Hypo- or Non-	40 (66.7)	59 (98.3)	
***Late phase***			0.000
Hyper-	1 (1.7)	0 (0)	
Iso-	16 (26.6)	1 (1.7)	
Hypo- or Non-	43 (71.7)	59 (98.3)	
***Enhancement duration#***	201.7 ± 146.4	33.5 ± 59.5	0.000
***Marked wash-out***	15 (25.0)	44 (73.3)	0.000

Note.---Unless otherwise indicated, data are number of nodules, with percentages in parentheses

*Statistical analysis using χ^2^ or Fisher’s exact test demonstrate differences between HAE and ICC

***#*** Data are means± standard deviations

*HAE* Hepatic alveolar echinococcosis; *ICC* Intrahepatic cholangiocarcinoma; US Ultrasound; *CEUS* Contrast enhanced ultrasound

**Table 2:** Characteristics on US in the training and testing set

Imaging Features on US	Training set (***n*** = 80)		Testing set (***n*** = 40)		P*
	HAE(*n* = 36)	ICC(*n* = 44)	HAE(*n* = 24)	ICC(*n* = 16)	
**Conventional US**					
***Location***					
Right lobe	30 (83.3)	24 (54.5)	20 (83.3)	9 (56.3)	0.252
Left lobe	6 (16.7)	20 (45.5)	4 (16.7)	7 (43.7)	0.393
***Lesion size***					
≤ 5.0 cm	14 (38.9)	17 (38.6)	8 (33.3)	7 (43.7)	0.755
> 5.0 cm	22 (61.1)	27 (61.4)	16 (66.7)	9 (56.3)	0.145
***Echogenicity***					
Hyper or Iso	5 (13.8)	3 (6.8)	5 (20.8)	4 (25.0)	1.000
Mixed	25 (69.4)	14 (31.8)	18 (75.0)	5 (31.3)	0.271
Hypo	6 (16.7)	27 (61.4)	1 (4.2)	7 (43.7)	1.000
***Margin***					
Clear	17 (47.2)	20 (45.5)	11 (45.8)	8 (50.0)	0.573
Unclear	19 (52.8)	24 (54.5)	13 (54.2)	8 (50.0)	0.287
***Bile duct dilatation***	1 (2.8)	16 (36.4)	1 (4.2)	5 (31.3)	0.462
***Pseudocystic appearance***	9 (25.0)	1 (2.3)	5 (20.8)	0 (0)	1.000
***Calcification***	16 (44.4)	0 (0)	13 (54.2)	0 (0)	NA
**CEUS**					
***Arterial phase***					
Hyper-	23 (63.9)	35 (79.5)	16 (66.7)	15 (93.8)	0.370
Iso-	5 (13.9)	8 (18.2)	5 (20.8)	1 (6.3)	0.141
Hypo- or Non-	8 (22.2)	1 (2.3)	3 (12.5)	0 (0)	1.000
***Enhancement pattern***					
Regular rim	33 (91.7)	4 (9.0)	20 (8.3)	2 (12.5)	1.000
Irregular rim	2 (5.6)	22 (50.0)	4 (16.7)	7 (43.8)	0.063
Heterogeneous	1 (2.7)	9 (20.5)	0 (0)	5 (31.2)	1.000
Homogeneous	0 (0)	9 (20.5)	0 (0)	2 (12.5)	NA
***Portal phase***					
Hyper-	1 (2.8)	0 (0)	0 (0)	0 (0)	NA
Iso-	9 (25.0)	0 (0)	10 (41.7)	1 (6.3)	1.000
Hypo- or Non-	26 (72.2)	44 (100)	14 (58.3)	15 (93.7)	0.3670
***Late phase***					
Hyper-	1 (2.8)	0 (0)	0 (0)	0 (0)	NA
Iso-	7 (19.4)	0 (0)	9 (37.5)	1 (6.3)	1.000
Hypo- or Non-	28 (77.8)	44 (100)	15 (62.5)	15 (93.7)	0.380
***Enhancement duration#***	197.4 ± 138.2	23.7 ± 16.2	205.9 ± 137.6	19.9 ± 5.3	
***Marked wash-out***	10 (27.8)	32 (72.7)	5 (20.8)	12 (75.0)	0.745

Note.---Unless otherwise indicated, data are number of nodules, with percentages in parentheses

***#*** Data are means± standard deviations

*Statistical analysis using χ^2^ or Fisher’s exact test demonstrate differences between the training set and testing set. *NA* Not available

*HAE* Hepatic alveolar echinococcosis; *ICC* Intrahepatic cholangiocarcinoma; *US* Ultrasound; *CEUS* Contrast enhanced ultrasound

### CEUS features for HAE and ICC

**Fig. 1:**
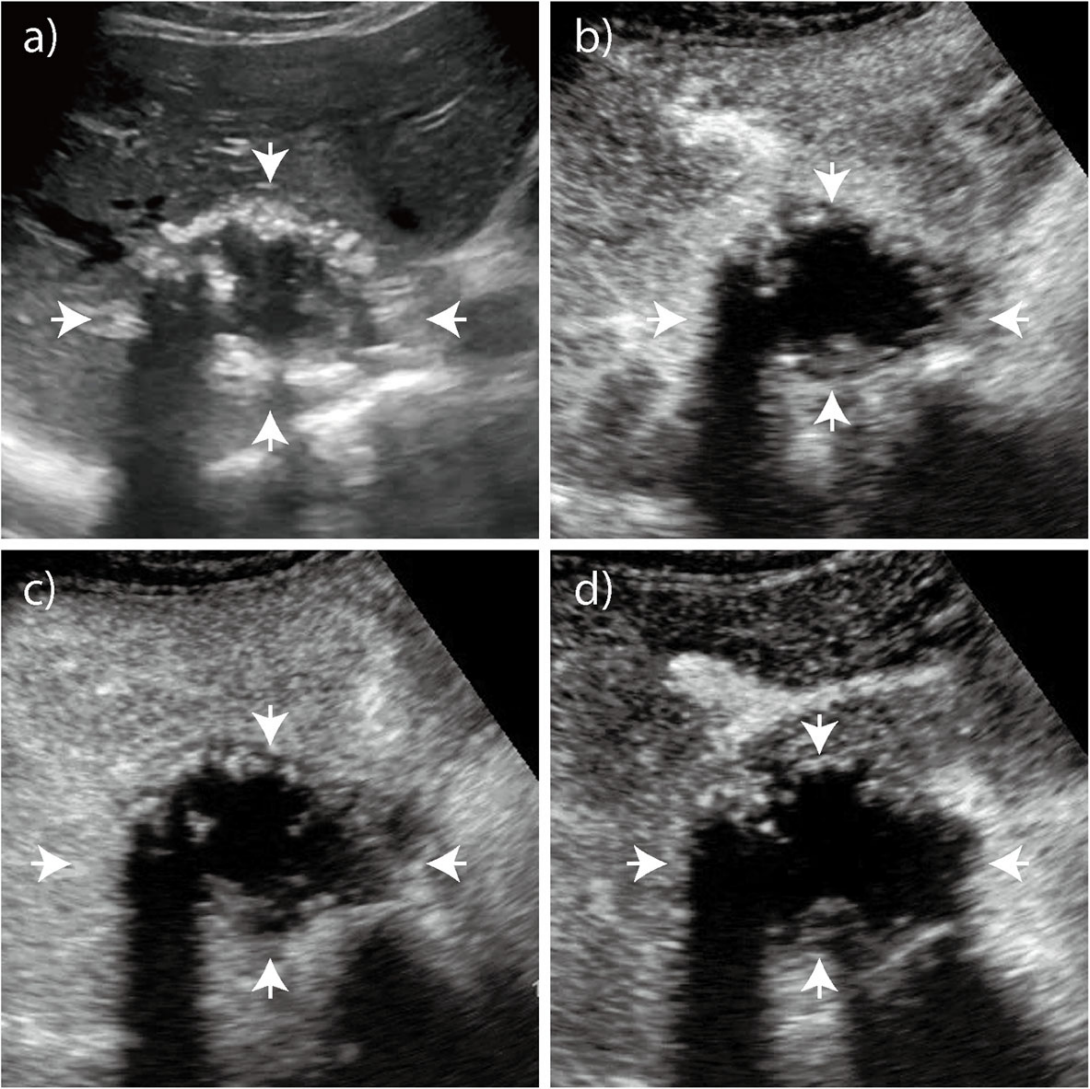
Hepatic alveolar echinococcosis (HAE). **a** Baseline ultrasound image shows a nodule with mixed echogenicity in the right lobe of the liver that is 5.8 cm in diameter. **b** On CEUS, arterial phase image obtained 19 s after administering the contrast agent shows a rim-like hyperenhancement of the lesion. **c** - **d** Portal and late phase images obtained 96 s and 129 s after administering the contrast agent, respectively. The nodule is hypoechoic with respect to the surrounding liver

**Fig. 2:**
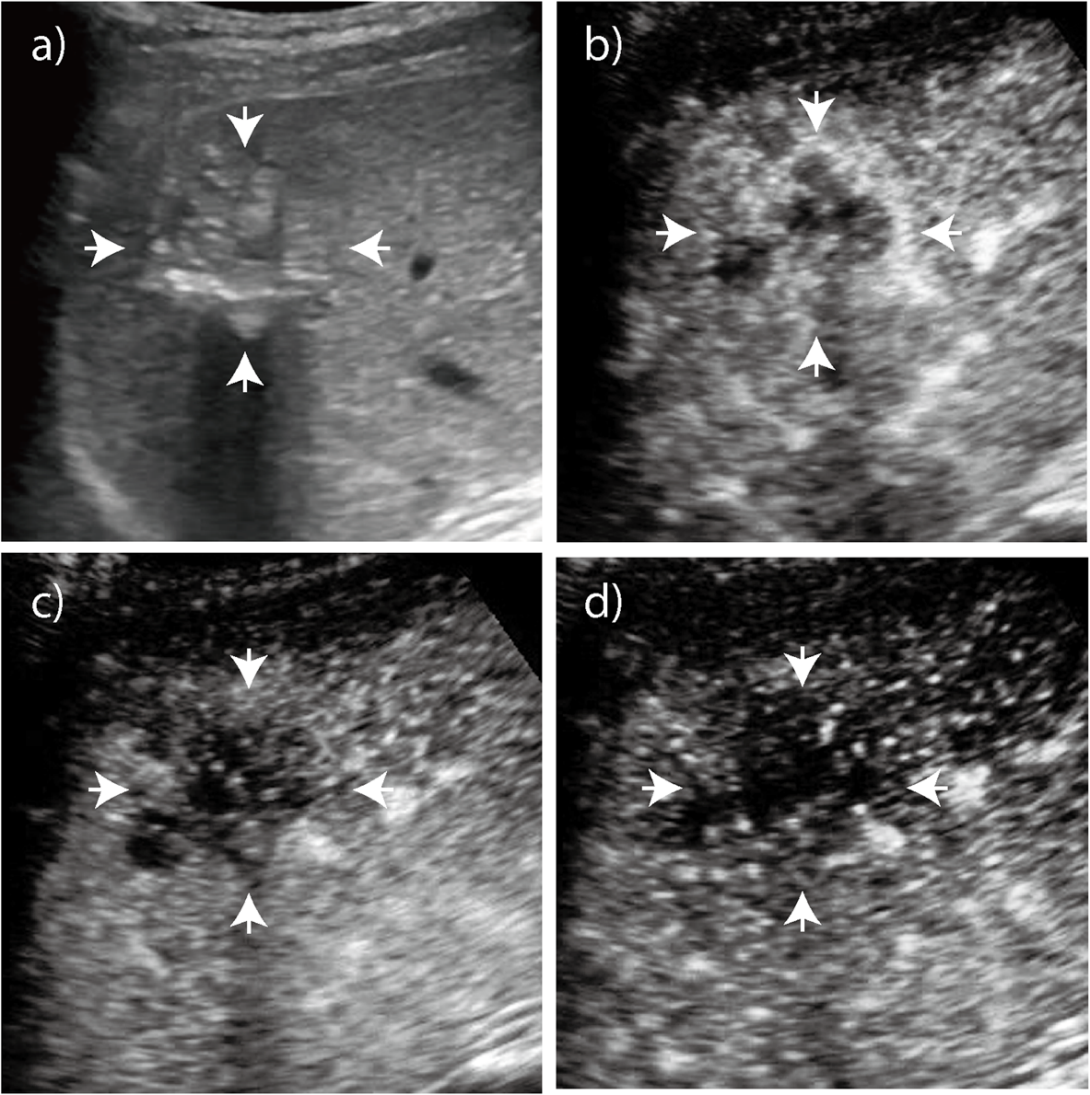
Hepatic alveolar echinococcosis (HAE). **a** Baseline ultrasound image shows a nodule with mixed echogenicity in the right lobe of the liver that is 5.0 cm in diameter. **b** On CEUS, arterial phase image obtained 15 s after administering the contrast agent shows diffuse heterogeneous hyperenhancement of the lesion. **c** - **d** Portal and late phase images obtained at 43 s and 131 s after administering the contrast agent, respectively. The nodule is hypoechoic with respect to the surrounding liver

**Fig. 3:**
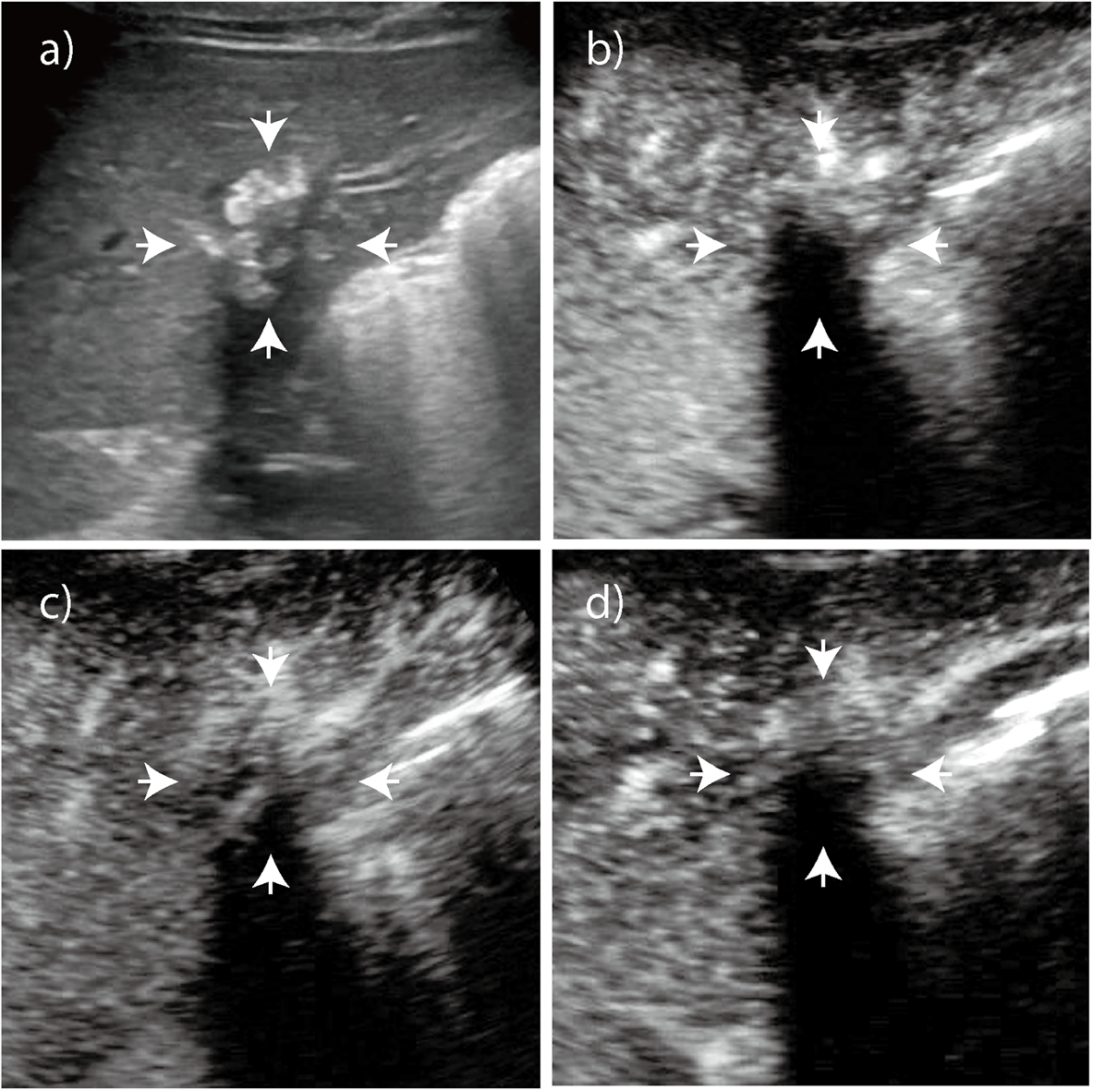
Hepatic alveolar echinococcosis (HAE). **a** Baseline ultrasound image shows a nodule with hyperechogenicity with macrocalcifications in the right lobe of the liver that is 2.9 cm in diameter. **b** - **d** On CEUS, no microbubble signals appear in the lesion in any of the three phases

**Fig. 4:**
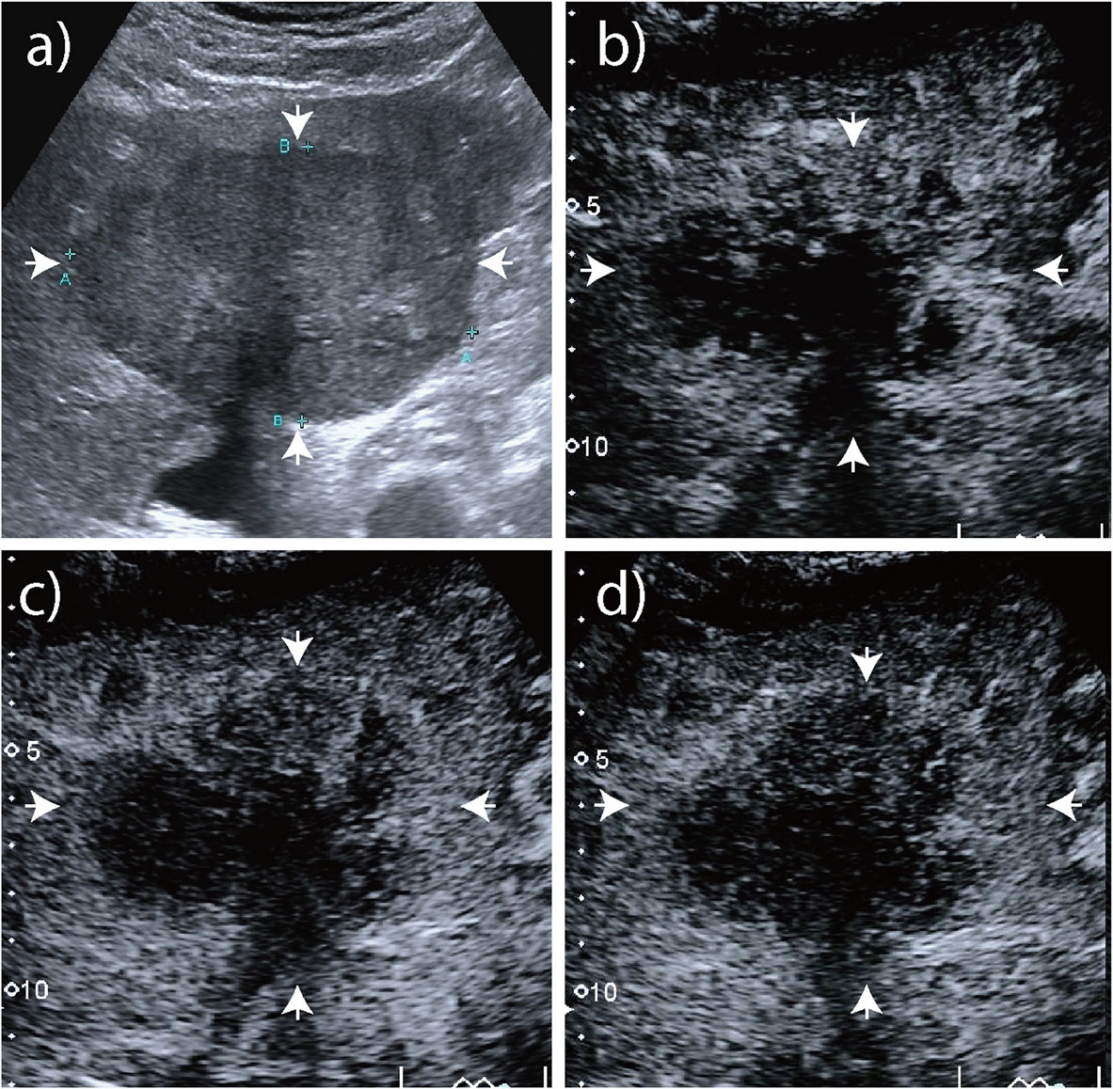
Intrahepatic cholangiocarcinoma (ICC). **a** Baseline ultrasound image shows a nodule with hypoechogenicity without macrocalcifications in the right lobe of the liver that is 8.7 cm in diameter. **b** In arterial phase on CEUS, the tumor appeared as irregular rim enhancement. **c** - **d** In portal and late phase, it faded out rapidly and showed marked washout

The enhancement patterns of 60 HAE lesions after an injection of SonoVue contrast agent are summarized in Table 1-2. The number of HAE lesions that showed hyper-, iso-, and hypoenhancement in the arterial phase were 39 (65.0%), 10 (16.7%), and 11 (18.3%), respectively; the number of ICC lesions that showed hyper-, iso-, and hypoenhancement were 50 (83.3%), 9 (15.0%), and 1 (1.7%), respectively (*P* = 0.008). Most HAE lesions exhibited regular peritumoral enhancement (*n* = 53, 88.3%). The areas of enhancement were primarily seen at the peripheral portions of the lesions and appeared as regular rim-like enhancement. The inner edge of the enhanced area was lumpy and strip-like and hardly extended to the central portion of the nodules (Figs. 1, 2, 3). However, irregular rims (*n* = 29, 48.3%) and heterogeneous or homogeneous enhancement (*n* = 25, 41.7%) were more commonly observed in ICC lesions than in HAE lesions (*P* = 0.000). In the portal and late phase, the enhanced areas in the HAE nodules faded out slowly (long enhancement duration: 201.7 s), but all ICC nodules faded out rapidly (short enhancement duration: 33.5 s). Forty lesions appeared with hypoenhancement, and 16 were isoenhanced in the late phase. While more (*n* = 59, 98.3%) ICC lesions appeared hypoenhanced in the late phase than HAE lesions, more (*n* = 44, 73.3%) ICC lesions showed marked washout than HAE lesions (*P* = 0.000) (Fig. 4).

### US score for diagnosing HAE

In the training set, LASSO regression analysis demonstrated that the selected conventional ultrasound and CEUS variables for differentiation were echogenicity, pseudocystic/calcification, bile duct dilatation, enhancement pattern, enhancement duration, and marked washout. In order to ensure reproductivity and easy access to clinical practice, a simplified scoring system is proposed, which gives assigned scores based on the coefficient of selected features (Table 3). For example, if an HAE lesion showed mixed echogenicity (1 points) with a pseudocystic appearance and calcifications (2 points) without bile duct dilatation (− 1 points) on conventional ultrasound, but the CEUS showed regular rim enhancement (1 point) with an enhancement duration of 150 s (1 point) and no marked washout (− 1 point), then the US total score would be 3. The mean US scores for HAE and ICC patients were 2.48 and 0.40, respectively, and the ROC analysis showed that the optimal cut-off value for differentiation was 1.0. If the US score for a patient was higher than this cut-off value, the diagnosis would be HAE; conversely, if the US score was lower than this cut-off value, the diagnosis would be ICC. Since the US score for this patient (3.0) was higher than the cut-off value of 1.0, the diagnosis was HAE.

**Table 3:** The Formation of an Equation for the US Score

Selected features	Coefficient	Signs	Assigned Score
Echogenicity	−0.11112163	Mixed-echogenicity	−1
Pseudocystic/calcification	1.12745008	Pseudocystic/calcification	1
Bile duct dilatation	−0.32246004	Bile duct dilatation	−1
Enhancement pattern	0.40994879	Regular rim	1
Enhancement duration	0.00372324	> 60s	1
Marked washout	−0.52185663	Marked washout	−1

*US* Ultrasound

### Diagnostic performance of the US score

In the training set, the diagnostic performance of the US score demonstrated that the sensitivity, specificity, LR+, LR- and AUC were 82.5, 86.4%, 6.05, 0.20 and 0.913, respectively. In the testing set, the sensitivity, specificity, LR+, LR- and AUC were 80.0, 81.3%, and 0.905, respectively. In the subgroup of lesions ≤5.0 cm, the sensitivity, specificity, LR+, LR- and AUC were 72.7, 91.7%, and 0.917, respectively. In the three cohorts, the diagnostic performances were excellent (all AUCs> 0.90, Table 4). The diagnostic performance of simple combined BUS and CEUS score was better than that of BUS or CEUS score alone (Table 4).

**Table 4:** Diagnostic Performance of BUS and/or CEUS for Differentiating between HAE and ICC

	Sensitivity^a^	95% CI^a^	Specificity^a^	95% CI^a^	AUC	95% CI	LR+	LR-
**BUS + CEUS score**								
Training set	82.5	67.2–92.7	86.4	72.6–94.8	0.913	0.832–0.964	6.05	0.20
Testing set	80.0	56.3–94.3	81.3	54.4–96.0	0.905	0.760–0.977	4.27	0.25
≤ 5.0 cm	72.7	49.8–89.3	91.7	73.0–99.0	0.912	0.791–0.975	8.73	0.30
**BUS score**								
Training set	67.5	50.9–81.4	100.0	92.0–100.0	0.886	0.798–0.945	NA	0.33
Testing set	60.0	36.1–80.9	100.0	79.4–100.0	0.925	0.786–0.986	NA	0.40
≤ 5.0 cm	45.5	24.4–67.8	100.0	85.8–100.0	0.875	0.744–0.954	NA	0.55
**CEUS score**								
Training set	82.5	67.2–92.7	65.9	50.1–79.5	0.777	0.673–0.861	2.42	0.27
Testing set	65.0	40.8–84.6	68.9	41.3–89.0	0.717	0.543–0.854	2.08	0.51
≤ 5.0 cm	72.7	49.8–89.3	75.0	53.3–90.2	0.773	0.625–0.883	2.91	0.36

^a^ Numbers are percentages

*HAE* Hepatic alveolar echinococcosis; *ICC* Intrahepatic cholangiocarcinoma; *CEUS* Contrast enhanced ultrasound; *AUC* Area under the curve; *PPV* Positive predictive value; *NPV* Negative predictive value

## Discussion

In our study, the typical conventional ultrasound and CEUS features of HAE such as hyper or mixed echogenicity with a pseudocystic appearance or calcifications on conventional ultrasound as well as regular rim enhancement and long enhancement duration on CEUS were combined; based on these features, we first developed a diagnostic US score for HAE that had an excellent accuracy of 95.0% and a perfect sensitivity of 100.0% in the testing cohort.

Conventional ultrasound could provide informative features for differentiating between HAE and ICC. In our study, most (88.3%) HAE lesions showed hyper or mixed echogenicity. A pseudocystic appearance or diffuse foci calcifications were observed in about 70% of the HAE lesions. However, ICC lesions commonly showed hypoechogenicity without cystic or necrotic areas [16]. Calcifications with/without acoustic shadowing were sometimes shown inside ICC lesions, but the foci calcifications were usually clustered with solid hypoechogenicity. Moreover, intrahepatic biliary dilatation was more commonly present in ICC than in HAE.

In recent years, CEUS has been introduced as a promising imaging technique for the diagnosis of HAE [9, 10, 17]. With the progress in contrast agents and contrast-specific imaging techniques, the parenchymal microvasculature of HAE lesions can be dynamically visualized on CEUS. Ehrhardt et al. compared the imaging features of HAE lesions on CEUS with those of fluorodeoxyglucose positron emission tomography (FDG-PET) [18]. The authors concluded that CEUS could assess the activity of HAE, and the findings with CEUS were consistent with the results of FDG-PET. In our study, 95.4% of the lesions were detected with enhancement, which correlates with active texture or an inflammatory reaction belt surrounding the lesion [10].

Most HAE lesions had regular rim enhancement and no enhancement in the center of lesion mass in the arterial and portal venous phases [17]. In our study, 84.8% of the lesions had this individual sign, which is similar to the percentage in previous reports (64–100%) [9, 17]. However, the rim enhancement pattern was also reported to be commonly detected in ICC lesions at a high rate of 68.5% [12]. In this study, we found that the rim enhancement of HAE lesions was regular and thin, which may be due to the enhancement of the alveolar wall. In contrast, the rim enhancement of ICC lesions was irregular and thicker than the linear rim of HAE lesions, which may be due to the infiltrative and abundant tumor cells at the periphery of the tumor.

In addition to the above typical CEUS feature, no enhancement, due to the vesicle structures, or diffuse heterogeneous hyperenhancement were also reported to be parts of the enhancement patterns for HAE lesions [9, 10]. These results are consistent with those of Cai et al’s study on evaluating 17 HAE lesions with CEUS [8]. Additionally, late or no obvious washout of the alveolar wall were suggestive features of HAE. However, diffuse heterogeneous enhancement was more common in ICC than in HAE. Other differences between ICC and HAE included the following: the enhancement of ICC lesions faded out more rapidly than that of HAE lesions, and most ICC lesions showed marked washout in the late phase.

The diagnostic performance of our US score for differentiating between these two entities demonstrated that its specificity was high (86.4%), and the AUC was excellent (0.9qe). The subgroup analysis for lesions smaller than 5.0 cm indicated that the differentiation performance of the US score was also high, with a sensitivity, specificity, and AUC of 72.7, 91.7%, and 0.912, respectively.

Our study had some limitations. First, the sample size of our study was relatively small, and ossification and hemangioma-like patterns in the HAE cases were rare. Consequently, the differentiation performance of the diagnostic score might be limited. Second, our study was retrospective, and further prospective studies are necessary, especially to explore if prognosis correlates with the different enhancement patterns of HAE. Future research about comparing the diagnostic performance of CEUS and CT/MRI will be also needed. Finally, CEUS may suffer from some of the same limitations as conventional sonography. For example, it may be difficult to detect lesions near the diaphragm dome or with a fatty liver background. Furthermore, the arterial phase lasts less than 1 min, so only one lesion or several lesions on the same plane can be observed with a single injection of contrast agent.

## Conclusion

The US score based on typical features from both conventional ultrasound and CEUS could accurately differentiate HAE from ICC. The enhancement pattern of HAE lesions on CEUS could provide informative advice for treatment decisions and be introduced as the standard modality for diagnosing HAE in patients who live in pastoral areas.

## Data Availability

The datasets generated and analyzed in this study are available upon reasonable request by the corresponding author. Email: lumd@live.com

## Abbreviations

AE: Alveolar echinococcosis
HAE: Hepatic alveolar echinococcosis
ICC: Intrahepatic cholangiocarcinoma
US: Ultrasound
CEUS: Contrast enhanced ultrasound
WHO-IWGE: World health organization informal working group on echinococcosis
SD: Standard deviation
LASSO: Least absolute shrinkage and selection operator
ROC: Receiver operating characteristic
AUC: Area under the curve
PPV: Positive predictive value
NPV: Negative predictive value
FDG-PET: Fluorodeoxyglucose positron emission tomography
CT: Computed tomography
MRI: Magnetic resonance imaging
